# Efficacy and Feasibility of an Osteopathic Intervention for Neurocognitive and Behavioral Symptoms Usually Associated With Fetal Alcohol Spectrum Disorder

**DOI:** 10.3389/fnbeh.2022.860223

**Published:** 2022-03-15

**Authors:** Ramon Cases-Solé, David Varillas-Delgado, Marta Astals-Vizcaino, Óscar García-Algar

**Affiliations:** ^1^Centre Osteopatia La Seu, Lleida, Spain; ^2^Department of Surgery and Medical-Surgical Specialties, Universitat de Barcelona, Barcelona, Spain; ^3^Faculty of Health Sciences, Universidad Francisco de Vitoria, Madrid, Spain; ^4^Department of Neonatology, Hospital Clínic-Maternitat, ICGON, BCNatal, Barcelona, Spain

**Keywords:** fetal alcohol spectrum disorder (FASD), prenatal alcohol exposure, osteopathic manipulative treatment, neurocognitive disorders, attention

## Abstract

The purpose of this study was to evaluate the efficacy and feasibility of a 4-week planned osteopathic manipulative treatment intervention on the improvement of neurocognitive and behavioral symptoms usually associated with fetal alcohol spectrum disorder. Thirty-two symptomatic children without fetal alcohol spectrum disorder aged 3–6 years with low level of attention from two schools and an osteopathic center were recruited in a prospective randomized pilot study in an osteopathic manipulative treatment group [osteopathic manipulative treatment (OMT)] or a control group (standard support measures). Neurocognitive maturity test results for attention (A), iconic memory (IM), spatial structuration (SS), and visual perception (VP) were recorded at baseline and post-intervention. No adverse effects were communicated and there were no dropouts. A significant increase in neurocognitive assessments was observed in children in the OMT group at post-treatment. Intergroup post-intervention statistical differences were found for A, SS, and IM were *p* = 0.005, *p* < 0.001, and *p* < 0.001, respectively; no differences were seen for VP (*p* = 0.097). This study shows that a 4-week osteopathic manipulative treatment intervention may be a feasible and effective therapeutic approach for neurocognitive and behavioral symptoms usually present in fetal alcohol spectrum disorder, justifying more studies on children affected by this condition.

## Introduction

Neurocognitive and behavioral symptoms are high incidence disabilities among children with FASD that affect the daily life of the patient, attention deficit being the most frequent (Weyrauch et al., 2017). Several systematic reviews underline the need for further research on the effectiveness of specific interventions aimed at early and individualized treatments of children with fetal alcohol spectrum disorders (FASDs), as well as new effective treatment strategies to improve neuropsychological symptoms in this population (Reid et al., 2015; Ordenewitz et al., 2021). According to the experience, osteopathy and its application through osteopathic manipulative treatment (OMT) may be an efficient therapeutic tool as an adjuvant treatment in FASDs. A systematic literature review was conducted on using PubMed, Medline and the Cochrane Library with the keywords “Fetal Alcohol Spectrum Disorder,” “Fetal Alcohol Syndrome,” “Osteopathic Manipulative Treatment,” “Neurocognitive Disorders,” and “Attention.” The reviewed literature indicates that children with FASDs may benefit from interventions when appropriately adapted to their neurodevelopmental disabilities (Petrenko, 2015) and may help improve their health-related quality of life (Stade et al., 2006). Similarly, there should be acceptance of the interventions by the patients and their families (Petrenko, 2015). This is very relevant for sensitive populations, such as the families with FASD members (Domeij et al., 2018; Flannigan et al., 2020; McLachlan et al., 2020; Pruner et al., 2020), particularly in communities such as ours, were prevalence of FASD of internationally adopted children is very high (Catalunya, 2019; Palacios et al., 2019).

Despite the global increase in the practice and specialization of pediatric osteopathy (International Alliance, 2020; DeMarsh et al., 2021; Schwerla et al., 2021), and its low-risk-profile (Hayes and Bezilla, 2006; DeMarsh et al., 2021), further research is needed to gather a body of evidence that could be used to recommend pediatric OMT under specific clinical conditions (DeMarsh et al., 2021). Thus, following recent recommendations in the literature (DeMarsh et al., 2021), assessment of viability and safety of OMT interventions in pediatric osteopathy are required before their use on specific population groups.

This preliminary study was designed on the assumption that the FASD population and their families will be receptive to experimental interventions (Stade et al., 2006; Domeij et al., 2018) and because of the lack of studies assessing the efficacy of OMTs on FASD-related neurocognitive and behavioral symptoms (Reid et al., 2015; Petrenko and Alto, 2017; Ordenewitz et al., 2021).

Positive effects of therapeutic interventions on neuropsychological symptoms in people with FASD have been shown, indicating that gains on attention (A) may be achieved, and generalize to other areas of functioning (Reid et al., 2015; Petrenko and Alto, 2017; Ordenewitz et al., 2021).

One of the purposes of osteopathy is to detect and correct somatic dysfunctions and their potential negative effects through manual contact by OMT. The results of research carried out to date suggest that OMT has anti-inflammatory (Standley and Meltzer, 2008; Licciardone et al., 2012; Degenhardt et al., 2017) and parasympathetic effects (Henley et al., 2008; Giles et al., 2013; Ruffini et al., 2015). Although specific metabolic and neurological alterations linked to the somatic dysfunction have been identified (Van Buskirk, 1990; Korr, 1991; Snider et al., 2011), the underlying physiological mechanisms remain under study (Tozzi, 2015; Tramontano et al., 2020; Roura et al., 2021). Moreover, there is evidence on the relation between the somatosensory system and neurological development processes, particularly in the areas of perception and cognition. Recent research has shown a dynamic interaction between the somatosensory system and a-related brain centers (Dockstader et al., 2010; Haegens et al., 2012; Wiesman and Wilson, 2020). Furthermore, other works have demonstrated effects on cortical plasticity after OMT interventions (Ponzo et al., 2018), as well as specific brain connectivity changes in sensorimotor, locomotor, and postural function networks, which suggests an alteration in the processing of information post-OMT (Tramontano et al., 2020).

Over the past years, there has been an increase in the number of publications on pediatric OMT, with additional evidence of its benefits in the field of neurological development disorders (DeMarsh et al., 2021). Nevertheless, further research is needed on the effectiveness of OMT in children (Parnell Prevost et al., 2019; DeMarsh et al., 2021).

Improvement of A in children and adolescents with attention deficit hyperactivity disorder (ADHD) has been seen (Accorsi et al., 2014), as well as positive effects in learning processes and infant neurological development (Frymann, 1976; Frymann et al., 1992). Social behavior and communication indexes ameliorated in a sample of children with autism (Bramati-Castellarin et al., 2016) as well as the mood, sleep, and limb function in children with cerebral palsy (Duncan et al., 2004). There is convincing evidence on the positive effect of OMT as adjuvant treatment in premature infants in neonate intensive care units (ICUs), e.g., decreased hospital stays and associated costs (Lanaro et al., 2017). Therefore, interventions such as an OMT may aid in neurocognitive and behavioral pediatric development including those to FASDs.

We hypothesized that standardized OMT aimed to correct individualized somatic dysfunctions would improve measures indicated by the Cumanin^®^ measuring test. The main primary objective of this pilot study was to evaluate the efficacy and feasibility of a 4-week planned OMT intervention delivered by a qualified pediatric osteopath, on Attention (A), iconic memory (IM), spatial structuration (SS) and visual perception (VP) in a group of children without FASD with low levels of A. The main secondary objective was to validate the intervention to apply it to FASD population in future studies.

## Materials and Methods

### Study Design

Prospective randomized pilot study.

### Patients

Children aged 3 to 6 years without a FASD diagnosis but with symptoms usually present in FASDs (Kodituwakku, 2009; Lange et al., 2017; Weyrauch et al., 2017; Maya-Enero et al., 2021) identified through a neuropsychological assessment referred from schools and an osteopathic center, were recruited between June 1 and July 17, 2020. Children with A and behavior problems according to their parents and/or teachers, following inattention criteria in the DSM-5 handbook were pre-selected (Battle, 2013). Reduced levels of A were recognized using the Neuropsychological Maturity Questionnaire for Children (Cumanin^®^) (Portellano Pérez et al., 2009) before the intervention during the recruitment process. Decreased levels of A were considered with scorings below the 50th percentile (*p* > 50) in the attention scale (Portellano Pérez et al., 2009). Due to the absence of previous studies, it was not possible to perform the estimated calculation of the sample size for this pilot study.

Children diagnosed with ADHD, or other neurological, genetic, and/or metabolic pathology, or receiving pharmacological treatment at the beginning of the intervention or had undergone OMT over the 12 months prior to the intervention, were excluded.

Informed consent to participate in the study was obtained from the parents/legal tutors, who also received written and verbal information on the design of the study and protocol. The Ethical Committee for Clinical Research Parc de Salut MAR (Barcelona, Spain) approved the study protocol (2016/7052/I), conducted according to the guidelines of the Declaration of Helsinki for Human Research of 1964 (last modified in 2013).

### Interventions

Two groups of children were defined: the OMT group (*n* = 16) children who received three OMT sessions over a 4-week period (one session every 2 weeks). Permuted-block randomization was used for treatment allocation. A research associate generated the random sequence using the Excel software. The control group (*n* = 16) were children who received standard support measures. Participants from both groups got the same tailored standard support learning measures at their schools, following the standard guidelines of educational intervention based on the creation of enabling environments and individualized support adapted to children with neurocognitive and behavioral symptoms, e.g., low level of A (Battle, 2013; Catalunya, 2019). Support measures received by the participants at school throughout the study period were not modified.

At the first intervention, each participant underwent a protocolized anamnesis and an osteopathic physical examination based on SOAP (Subjective, Objective, Plan, Assessment) notes and exam forms (Sleszynski et al., 1999; Sleszynski and Glonek, 2005). Somatic dysfunctions were detected by physical examination, based on tissue texture changes, asymmetry, limitation in normal range of motion, and tissue tenderness parameters (TART), which guided the osteopathic evaluation and OMT intervention. The parameters of somatic dysfunctions were described by the position and motion of a body part as determined by palpation.

Using OMT techniques, the identified somatic dysfunctions were corrected one by one in the whole body (Tramontano et al., 2020). The following approaches were used: balanced ligamentous techniques, balanced membranous techniques/osteopathy in the cranial field, and facilitated positional release techniques (Johnson and Kurtz, 2003). An osteopathic physical examination and an OMT intervention were performed in each session to assess and correct somatic dysfunctions. The time allocated for the first session was 50 min, and the next two 30 min each.

To improve adherence and reduce performance bias, participants were assigned the OMT the same day every week. Reminder and confirmation calls were made to families 24 h before each scheduled intervention and before the pre-/post-tests.

A qualified pediatric osteopath, with a master’s degree in Osteopathy, following the recommendations of the European Standard UNE-EN 16686 (16686:2015), and a postgraduate specialization in Pediatric Osteopathy, carried out the OMT interventions. A qualified psychologist performed the neuropsychological pre-/post-tests to all participants at baseline and at conclusion of the intervention (the day after the last OMT session). Specific and general recommendations of each questionnaire were followed. Pre- and post-tests took between 20 and 30 min each (Portellano Pérez et al., 2009).

### Outcome Variables

The primary outcome for validating and assessing the OMT was the percentage of patients who completed the intervention and showed statistically significant differences in the individually administered Cumanin^®^ measuring test, which includes neuropsychological maturity scales that allow to determine the centile values for A, IM, SS, and VP (Portellano Pérez et al., 2009). Investigators who performed and assessed the OMT were blinded to patient random allocation.

#### Description and Neurofunctional Significance of the Neurocognitive Scales

Attention (A): 20 items – the aim was to identify and mark 20 geometrical figures identical to the proposed model (a square) shown among 100 figures, 80 of which were distractors and 20 squares identical to the model. The test was carried out for 30 sec and the correct answers (correctly crossed-out squares) and errors (other incorrectly crossed-out figures) were noted, although only the number of correctly crossed-out figures was taken into account. Maximum score = 20; minimum = 0. This assesses structures that are involved with A processes, particularly reticular formation and prefrontal cortex. The right cerebral hemisphere is dominant in A control (Portellano Pérez et al., 2009).

Iconic memory (IM): 10 items – the child had to memorize 10 simple drawings of objects for 1 min. Then, the child had to say the name of the drawings he remembered, in a period of 90 sec. The child got 1 point for each well-remembered object. It was not considered if child said an incorrect object. Maximum score = 10; minimum = 0. Immediate memory is related to structures such as the hippocampus, parietal cortex, and amygdala. This scale evaluates right hemisphere function (Portellano Pérez et al., 2009).

Spatial structuration (SS): 15 items – the child had to perform increasingly difficult spatial orientation activities via psychomotor (11 items) and graphomotor responses (4 items). Maximum score = 15; minimum = 0). Essentially, this is related with association centers at the parietal-temporal-occipital cortex, in charge of spatial representation on the Penfield sensory homunculus at the parietal cortex (Portellano Pérez et al., 2009).

Visual perception (VP): the child had to reproduce 15 items geometrical designs of increasing difficulty. Each correctly drawn figure was valued with 1 point. The test ended if the child made 4 consecutive drawings wrong. Maximum score = 15; minimum = 0. Secondary visual areas and associative areas on the occipital lobe mediate this, as well as the mnemonic function, which is mediated by deeper areas of the temporal cortex. The frontal cortex is also involved, along with various motor-decision centers of the brain (Portellano Pérez et al., 2009).

Each scale allows scores to be recorded, the interpretation of which is made by converting these raw scores into centile scales, which are differentiated into five age groups in months. Scores below normal are considered to be centiles from 20 to 40, with scores below the 20th centile being considered very low (Portellano Pérez et al., 2009).

### Statistical Analysis

All statistical analyses were carried out using the Statistical Package for the Social Sciences (SPSS) v.21.0 for Windows (IBM Corp. Released 2012. IBM SPSS Statistics for Windows, Version 21.0. Armonk, NY, United States: IBM Corp). Categorical variables were evaluated using frequencies and percentages and quantitative variables with means and standard deviations that included maximum and minimum values (range). Distribution of the data was evaluated using Shapiro-Wilk test. Comparisons at various time intervals within each group were analyzed using Friedman’s test and, if statistical significance was detected, multiple comparisons were carried out using Wilcoxon’s sign rank test. Categorical variables were analyzed using the Pearson’s chi-square (χ^2^) test. Groups were compared with Kruskal-Wallis test complemented by the Bonferroni correction. Cohen’s d was calculated to evaluate effect sizes. *P* values < 0.05 were considered statistically significant.

## Results

Thirty-two participants (*n* = 32) without FASD were included in this study, 16 in the OMT group and 16 in the control group. Gender ratio (male: female) was 17:15; 10:6 for the OMT group and 7:9 for control group. No adverse effects were communicated and none of the participants dropped out. Demographic characteristics of children from both groups are shown in Table 1.

**TABLE 1:** Baseline characteristics of the study groups.

	Control group (*n* = 16)	OMT group (*n* = 16)	*P* value
Boys/girls	7/9	10/6	0.288
Age at study entry (months), mean (SD)	48.81 (10.001)	54.75 (9.370)	0.093
Attention, median [IQR]	27.50 [15.00–35.00]	20.00 [5.00–35.00]	0.152
Iconic memory, median [IQR]	75.00 [61.25–80.00]	75.00 [60.00–80.00]	0.931
Spatial structuration, median [IQR]	65.00 [60.00–70.00]	80.00 [35.00–93.75]	0.085
Visual perception, median [IQR]	45.00 [40.00–75.00]	57.50 [40.00–78.75]	0.212

*IQR, interquartile range; OMT, osteopathic manipulative treatment; SD, standard deviation.*

Average duration of the interventions was as follows: anamnesis -1st session- 16.25 min, exploration, 10.83 min, and treatment, 10.42 min. Average number of somatic dysfunctions (SD) were [most prevalent: cranial (30.0%), diaphragm (17.1%), and cervical area (12.8%)] per participant at baseline was 4, dropping to 1.5 at the last OMT session. Percentages of the used approaches were as follows: balanced ligamentous techniques (61.4%), balanced membranous techniques/osteopathy in the cranial field (30%), and facilitated positional release (8.6%).

### Participant Flow

Forty-three (*n* = 43) children were pre-selected; eight were excluded because of a percentile above 50 in the A scale. Thirty-five candidates (*n* = 35) were enrolled, of whom three were excluded for not meeting the inclusion criteria, i.e., had received OMT treatment over the past 12 months (*n* = 2) and undergoing pharmacological treatment (*n* = 1). Thirty-two participants were finally included in the study, 16 randomly allocated to the OMT group and 16 to the control group. One osteopath from a single osteopathic center delivered the OMTs. Standard support measures were applied at school (*n* = 12). Statistical analyses were performed including the 32 participants. All completed the treatment and there were no dropouts.

Statistically significant differences were observed for A in the control group (*p* = 0.027) and in the OMT group (*p* = 0.031) (Table 2) following Friedman’s test; similarly, differences (*p* < 0.001) were found for SS in the control and OMT groups (Table 3). Statistically significant differences were seen for VP only in OMT group (*p* = 0.019) (Table 4); no statistically significant pre-post results were seen for IM in the control group, contrary to what was observed in the treatment group (*p* < 0.001) (Table 5).

**TABLE 2:** Neuropsychological maturity test average centile scores for attention.

	Attention			
	Pre-intervention, median [IQR]	Post-intervention, median [IQR]	Effect size (within group)	*P* value
Control	27.50 [15.00–35.00]	17.50 [15.00–23.75]	−0.58	0.027
OMT	20.00 [5.00–35.00]	30.00 [20.00–43.75]	0.34	0.031

*IQR, interquartile range; OMT, osteopathic manipulative treatment.*

**TABLE 3:** Neuropsychological maturity test average centile scores for spatial structuration.

	Spatial structuration			
	Pre-intervention, median [IQR]	Post-intervention, median [IQR]	Effect size (within group)	*P* value
Control	65.00 [60.00–70.00]	30.00 [21.25–50.00]	−1.80	<0.001
OMT	80.00 [35.00–93.75]	57.50 [30.00–83.75]	−0.68	<0.001

*IQR, interquartile range; OMT, osteopathic manipulative treatment.*

**TABLE 4:** Neuropsychological maturity test average centile scores for visual perception.

	Visual perception			
	Pre-intervention, median [IQR]	Post-intervention, median [IQR]	Effect size (within group)	*P* value
Control	45.00 [40.00–75.00]	45.00 [26.25–48.75]	−0.31	0.184
OMT	57.50 [40.00–78.75]	47.50 [35.00–70.00]	−0.42	0.019

*IQR, interquartile range; OMT, osteopathic manipulative treatment.*

**TABLE 5:** Neuropsychological maturity test average centile scores for iconic memory.

	Iconic memory			
	Pre-intervention, median [IQR]	Post-intervention, median [IQR]	Effect size (within group)	*P* value
Control	75.00 [61.25–80.00]	65.00 [60.00–80.00]	−0.32	0.117
OMT	75.00 [60.00–80.00]	90.00 [80.00–95.00]	1.22	<0.001

*IQR, interquartile range; OMT, osteopathic manipulative treatment.*

Post-treatment statistically significant differences were found for A, SS, and IM between the treatment and control groups (*p* = 0.005, *p* < 0.001, and *p* < 0.001, respectively). This was not the case for VP, for which no statistical differences (*p* = 0.097) were determined by Kruskal-Wallis test complemented by the Bonferroni correction (Table 6).

**TABLE 6:** Post-treatment average score differences in the two study groups.

	OMT group, median difference [IQR]	Control group, median difference [IQR]	Effect size (between group)	*P* value
Attention	10.00 [7.75–15.00]	−10.00 [−11.25−0.00]	0.62	0.005
Iconic memory	15.00 [15.00–20.00]	−10.00 [−13.00- −4.00]	0.86	<0.001
Spatial structuration	−22.50 [−29.00- −5.00]	−35.00 [−38.75- −20.00]	0.79	<0.001
Visual perception	−10.00 [−18.75- −5.00]	0.00 [−7.00–5.50]	−0.51	0.097

*IQR, interquartile range; OMT, osteopathic manipulative treatment.*

No relevant adverse events or side effects were communicated.

## Discussion

The objective of this study was to evaluate the efficacy and feasibility of an OMT intervention on neurocognitive and behavioral symptoms commonly present in FASD (Kodituwakku, 2009; Weyrauch et al., 2017; Maya-Enero et al., 2021), and validate the intervention to apply it to FASD population in the future.

This work shows that a 4-week OMT plan, administered by a qualified pediatric osteopath, is a feasible therapeutic approach for children aged 3–6 years who exhibit neurocognitive and behavioral symptoms usually present in FASD, such as attention deficit (Kodituwakku, 2009; Lange et al., 2017; Weyrauch et al., 2017; Maya-Enero et al., 2021), effectively improving A, SS, and IM, but not VP.

The development of perception and cognition is linked to the somatosensory system (Dockstader et al., 2010; Haegens et al., 2012; Wiesman and Wilson, 2020) and the potential effects of somatic dysfunctions (Frymann, 1976; Frymann et al., 1992; Accorsi et al., 2014). The sociodemographic profile of the FASD population in our community (Catalunya, 2019) is characterized by a high index of FASD children who have been adopted from countries of Eastern Europe, with high prevalence of special needs (Palacios et al., 2019) and may thus be susceptible to experimental interventions. Thus, in our opinion, prior validation of any new therapeutic intervention aimed at this population should be a priority. Therefore, the current study evaluates cases with neurocognitive and behavioral symptomatology without a FASD diagnosis (Maya-Enero et al., 2021). The aim was to assess the feasibility of an OMT intervention that could be used for FASD individuals. Our results, as a preliminary intervention tool, show that OMT can be a valid approach for treating neurocognitive and behavioral symptoms usually present in FASDs (Kodituwakku, 2009; Lange et al., 2017; Weyrauch et al., 2017; Maya-Enero et al., 2021).

Research evidence indicates that gains in A can be achieved in FASD populations (Reid et al., 2015; Petrenko and Alto, 2017; Ordenewitz et al., 2021).

In our review of the literature we did not find studies evaluating OMT interventions on cases with neurocognitive and behavioral symptoms usually associated with FASD (Reid et al., 2015; Petrenko and Alto, 2017; Ordenewitz et al., 2021). Moreover, there is lack of relevant studies measuring the efficacy of OMT on neuropsychological development. Accorsi et al. suggest that OMT may improve selective and sustained A performances in children and adolescents with ADHD (Accorsi et al., 2014), although this should be further investigated.

Absence of adverse effects in our study may be due to the lower incidence of adverse events immediately after the OMT in comparison to other manual medical disciplines (Degenhardt et al., 2018), while the gentle, non-invasive, tailored health care approach of OMT may have helped maintain patient adherence (World Health Organization, 2012). Possibly, protocolized osteopathic anamnesis and examination, the training and experience of the care providers, and supervision of the procedures, are additional factors that may have contributed to the success of the interventions. Families of children affected by FASD show great interest in receiving care and treatment (Lange et al., 2018; Flannigan et al., 2020), which may help maintain a low dropout rate in future interventions with this population. Further research is required to assess OMT efficacy and patient’s safety (Degenhardt et al., 2018).

In this study, we show positive post-OMT outcomes on a defined population, significant in three of the four assessed variables. These results may be because OMT interventions have on somatic dysfunctions, which consequently reduce the potential negative consequences on perceptual and cognitive development (Frymann, 1976; Frymann et al., 1992; Tozzi, 2015). More research is needed to assess the effect of OMT interventions in children younger than 6 years with low levels of A. Post-treatment results show a favorable effect of overall neuropsychological development OMTs toward the negative evolution of these variables over time. Although the characteristics of this study do not allow to draw additional conclusions, the results suggest the need of more in-depth studies on the evolution of overall neuropsychological development in pre-school children as stated by Sjöwall et al. (2017) study. However, early neuropsychological deficits may be identified and have predictive value in future development of ADHD symptoms and subsequent academic performance (Sjöwall et al., 2017). Still, the small sample size and duration of the study limit any conclusion. More studies with larger samples and longer study duration are recommended.

The average number of somatic dysfunctions per participant at baseline was 4 [most prevalent: cranial (30%), diaphragm (17.1%), and cervical area (12.8%)] and 1.5 at the last session. OMT interventions may explain the observed positive results on somatic dysfunctions. These results seem to corroborate data from previous works (Accorsi et al., 2014), although the characteristics of our study limit further comparisons. The different levels of improvement may be explained by the various development processes and maturation pathways of each measured variable (Portellano Pérez et al., 2009), suggesting that somatic dysfunctions and OMT interventions may have distinct effects on each process. Additional research is needed to deepen into the mechanisms of OMT on somatic dysfunctions (Tozzi, 2015). Brain plasticity and neurodevelopment mechanisms present during the first stages of life may explain the positive effects observed in our work despite the short duration of the study (Portellano Pérez et al., 2009; Lange et al., 2017). This supports the importance of early interventions in neurocognitive and behavioral disorders (Portellano Pérez et al., 2009; Reid et al., 2015; Petrenko and Alto, 2017; Ordenewitz et al., 2021).

Despite the relevant findings, this study has some limitations. This is a pilot study showing a favorable effect of OMT on children between 3 and 6 years of age with attention deficits. More research is needed to assess whether this intervention may be able to help all children with attention problems, including those with FASD. Attention deficit in our study population may have a different etiology than that of the FASD population, which may lead to distinct post-intervention results and conclusions in comparison to those observed in a FASD population (Glass et al., 2013; Boseck et al., 2015). In cases of PAE, the impact of combined genetic and epigenetic factors throughout pre- and postnatal development, makes it difficult to establish a specific neuropsychological profile (Mattson et al., 2019; Maya-Enero et al., 2021) or determine its progression over time (Weyrauch et al., 2017).

Other limitations are the small sample size, which restricts the assessment of efficacy, and age of participants. In the latter, OMT on individuals aged 3 to 6 years would enable to intervene in early neural development and deliver the intervention during early neurodevelopmental difficulties. However, the number and variety of neurocognitive and behavioral evaluation tools for children under 6 years is scarce (Portellano Pérez et al., 2009; Coles et al., 2021). The short length of the study is a limitation to objectify improvements in neuropsychological development. Moreover, the capacity children have for learning and remembering the tests may be a bias in terms of evaluation (Portellano Pérez et al., 2009). To homogenize our sample based on attention deficit, we carried out an assessment of A using the scale of the Cumanin^®^ neuropsychological battery throughout 4 weeks before the intervention, a factor that may increase the recall bias in the variable. The above-mentioned limitations can be reduced by increasing the size of the sample and study duration, as well as an extended follow-up period beyond the post-treatment period (Reid et al., 2015), as this would allow to determine if the achieved results are maintained over time. Moreover, other assessment tools can be used before the intervention during the recruitment period to reduce the recall bias for this variable.

Cumanin^®^ is a neuropsychological assessment instrument validated in Spain for children between 36 and 78 months of age, widely used in Spain and other Spanish speaking countries (Urzúa et al., 2010; Ávila Matamoros, 2012; Salvador-Cruz et al., 2019). This means that the results may be not reproducible in samples from populations from different countries. The reliability of the questionnaire is considered acceptable and supported by a study that includes a sample of 803 participants (Portellano Pérez et al., 2009). Thus, four specific scales of the Cumanin^®^ questionnaire were used. Although the scales have been designed to measure each variable independently, using the scales separately may imply a potential bias. This was compensated by strictly following the instructions and steps described for the evaluation (Portellano Pérez et al., 2009). During the drafting of this manuscript, a new version of the Cumanin questionnaire was published (Cumanin^®^-2) (Portellano-Pérez et al., 2021), an extended and updated version of Cumanin^®^ for the neuropsychological assessment of children. Our study did not aim to assess and analyze the overall neuropsychological status of the participants, but to evaluate the selected variables and determine their evolution over time. Therefore, the used assessment tools in this work retain their validity regarding pre- and post-intervention assessments. Moreover, in future interventions involving FASD populations within this age range, the use of Cumanin^®^-2 should be considered, because to date, there is no references in the literature that describe the clinical significance of the changes in the measurements of the scales used.

Participants and their families were not blinded to the OMT intervention, and no sham-intervention or placebo treatment was offered due to the lack of standard guidelines for OMT use (Cerritelli et al., 2016). Therefore, a placebo effect should be considered in the current study, which can be overcome by a homogeneous well-reported sham therapy applied to a third group, using a wait-listed control group or a crossover study design in future studies. Due to its importance, the creation of standard well-reported placebo treatments and their application in OMT clinical trials should be considered in further works (Cerritelli et al., 2016). In addition, the lack of a predetermined treatment protocol limits the generalizability of the results. Moreover, this factor allows the intervention on the FASD population to be tailored to the patient’s profile and symptoms, as noted in a recent systematic review (Ordenewitz et al., 2021). This is a common obstacle in the field of manual medicine that can be minimized by applying standardized procedures (Alvarez et al., 2016). Following anamnesis and exploration protocols, discussion and supervision of the procedures among several professionals, and specific training and education of care providers, were measures adopted to minimize this limitation.

Although the characteristics of this study do not allow drawing further conclusions, our results suggest the need for further studies on certain clinical presentations characterized by deficits in neurocognitive and behavioral development, a field explored by Frymann et al. (1992) several years ago (Frymann, 1976).

## Conclusion

Our study provides important data supporting the need for more rigorous trials. Statistically significant post-intervention differences between treatment and control groups were observed for A, SS, and IM; no differences were seen for VP. Significative changes in A and IM were observed in the treatment group. No adverse effects were communicated and none of the participants dropped out. Our results justify the design of a controlled clinical study to evaluate the feasibility and efficacy of OMT interventions in FASD populations with larger samples, extended follow-up periods, and a sham therapy to a third group of participants.

## Data Availability Statement

The original contributions presented in the study are included in the article/supplementary material, further inquiries can be directed to the corresponding author.

## Ethics Statement

The studies involving human participants were reviewed and approved by the Ethical Committee for Clinical Research Parc de Salut Mar, Barcelona, Spain (2016/7052/I). Written informed consent to participate in this study was provided by the participants’ legal guardian/next of kin.

## Author Contributions

RC-S conceived the experiments. RC-S, MA-V, and ÓG-A designed and performed the experiments. DV-D analyzed the data. RC-S, DV-D, MA-V, and ÓG-A wrote the manuscript. All authors contributed to the article and approved the submitted version.

## Conflict of Interest

The authors declare that the research was conducted in the absence of any commercial or financial relationships that could be construed as a potential conflict of interest.

## Publisher’s Note

All claims expressed in this article are solely those of the authors and do not necessarily represent those of their affiliated organizations, or those of the publisher, the editors and the reviewers. Any product that may be evaluated in this article, or claim that may be made by its manufacturer, is not guaranteed or endorsed by the publisher.

## References

[B1] Accorsi A. Lucci C. Di Mattia L. Granchelli C. Barlafante G. Fini F. (2014). Effect of osteopathic manipulative therapy in the attentive performance of children with attention-deficit/hyperactivity disorder. *J. Am. Osteopath. Assoc.* 114 374–381. 10.7556/jaoa.2014.074 24778002

[B2] Alvarez G. Cerritelli F. Urrutia G. (2016). Using the template for intervention description and replication (TIDieR) as a tool for improving the design and reporting of manual therapy interventions. *Man Ther.* 24 85–89. 10.1016/j.math.2016.03.004 27029717

[B3] Ávila Matamoros A. M. (2012). Adaptación del cuestionario de madurez neuropsicológica infantil Cumanin de Portellano. *Rev. Iberoam. Psicol.* 5 91–100

[B4] Battle D. E. (2013). Diagnostic and statistical manual of mental disorders (DSM). *Codas* 25 191–192. 24413388 10.1590/s2317-17822013000200017

[B5] Boseck J. J. Davis A. S. Cassady J. C. Finch W. H. Gelder B. C. (2015). Cognitive and adaptive skill profile differences in children with attention-deficit hyperactivity disorder with and without comorbid fetal alcohol spectrum disorder. *Appl. Neuropsychol. Child* 4 230–236. 10.1080/21622965.2013.877392 25318015

[B6] Bramati-Castellarin I. Patel V. B. Drysdale I. P. (2016). Repeat-measures longitudinal study evaluating behavioural and gastrointestinal symptoms in children with autism before, during and after visceral osteopathic technique (VOT). *J. Bodyw. Mov. Ther.* 20 461–470. 10.1016/j.jbmt.2016.01.001 27634066

[B7] Catalunya (2019). *Informe Prevalença.* Available online at: https://dretssocials.gencat.cat/web/.content/03ambits_tematics/01acollimentsiadopcions/destacats_dreta/Informe-Final_Projecte-prevalenca-TEAF-nens-adoptats-Catalunya_ok.pdf

[B8] Cerritelli F. Ruffini N. Lacorte E. Vanacore N. (2016). Osteopathic manipulative treatment in neurological diseases: systematic review of the literature. *J. Neurol. Sci.* 369 333–341. 10.1016/j.jns.2016.08.062 27653920

[B9] Coles C. D. Kable J. A. Granovska I. V. Pashtepa A. O. Wertelecki W. Chambers C. D. (2021). Measurement of neurodevelopmental effects of prenatal alcohol exposure in ukrainian preschool children. *Child Neuropsychol.* 27 1088–1103. 10.1080/09297049.2021.1919298 33982636 PMC9205268

[B10] Degenhardt B. F. Johnson J. C. Brooks W. J. Norman L. (2018). Characterizing adverse events reported immediately after osteopathic manipulative treatment. *J. Am. Osteopath. Assoc.* 118 141–149. 10.7556/jaoa.2018.033 29480914

[B11] Degenhardt B. F. Johnson J. C. Fossum C. Andicochea C. T. Stuart M. K. (2017). Changes in cytokines, sensory tests, and self-reported pain levels after manual treatment of low back pain. *Clin. Spine Surg.* 30 E690–E701. 10.1097/BSD.0000000000000231 28632555

[B12] DeMarsh S. Huntzinger A. Gehred A. Stanek J. R. Kemper K. J. Belsky J. A. (2021). Pediatric osteopathic manipulative medicine: a scoping review. *Pediatrics* 147:e2020016162. 10.1542/peds.2020-016162 33500321

[B13] Dockstader C. Cheyne D. Tannock R. (2010). Cortical dynamics of selective attention to somatosensory events. *Neuroimage* 49 1777–1785. 10.1016/j.neuroimage.2009.09.035 19781649

[B14] Domeij H. Fahlström G. Bertilsson G. Hultcrantz M. Munthe-Kaas H. Gordh C. N. (2018). Experiences of living with fetal alcohol spectrum disorders: a systematic review and synthesis of qualitative data. *Dev. Med. Child Neurol.* 60 741–752. 10.1111/dmcn.13696 29479676

[B15] Duncan B. Barton L. Edmonds D. Blashill B. M. (2004). Parental perceptions of the therapeutic effect from osteopathic manipulation or acupuncture in children with spastic cerebral palsy. *Clin. Pediatr.* 43 349–353. 10.1177/000992280404300406 15118778

[B16] Flannigan K. Coons-Harding K. D. Anderson T. Wolfson L. Campbell A. Mela M. (2020). A systematic review of interventions to improve mental health and substance use outcomes for individuals with prenatal alcohol exposure and fetal alcohol spectrum disorder. *Alcohol. Clin. Exp. Res.* 44 2401–2430. 10.1111/acer.14490 33119894 PMC7839542

[B17] Frymann V. M. (1976). Learning difficulties of children viewed in the light of the osteopathic concept. *J. Am. Osteopath. Assoc.* 76 46–61. 1048966

[B18] Frymann V. M. Carney R. E. Springall P. (1992). Effect of osteopathic medical management on neurologic development in children. *J. Am. Osteopath. Assoc.* 92 729–744. 10.7556/jaoa.1992.92.6.729 1377192

[B19] Giles P. D. Hensel K. L. Pacchia C. F. Smith M. L. (2013). Suboccipital decompression enhances heart rate variability indices of cardiac control in healthy subjects. *J. Altern. Complement. Med.* 19 92–96. 10.1089/acm.2011.0031 22994907 PMC3576914

[B20] Glass L. Ware A. L. Crocker N. Deweese B. N. Coles C. D. Kable J. A. (2013). Neuropsychological deficits associated with heavy prenatal alcohol exposure are not exacerbated by ADHD. *Neuropsychology* 27 713–724. 10.1037/a0033994 24040921 PMC3898510

[B21] Haegens S. Luther L. Jensen O. (2012). Somatosensory anticipatory alpha activity increases to suppress distracting input. *J. Cogn. Neurosci.* 24 677–685. 10.1162/jocn_a_00164 22066587

[B22] Hayes N. M. Bezilla T. A. (2006). Incidence of iatrogenesis associated with osteopathic manipulative treatment of pediatric patients. *J. Am. Osteopath. Assoc.* 106 605–608. 17122030

[B23] Henley C. E. Ivins D. Mills M. Wen F. K. Benjamin B. A. (2008). Osteopathic manipulative treatment and its relationship to autonomic nervous system activity as demonstrated by heart rate variability: a repeated measures study. *Osteopath. Med. Prim. Care* 2:7. 10.1186/1750-4732-2-7 18534024 PMC2442110

[B24] International Alliance (2020). *The OIA Global Report: Global Review of Osteopathic Medicine and Osteopathy.* Available online at: https://oialliance.org/the-oia-global-report-global-review-of-osteopathic-medicine-and-osteopathy-2020/

[B25] Johnson S. M. Kurtz M. E. (2003). Osteopathic manipulative treatment techniques preferred by contemporary osteopathic physicians. *J. Am. Osteopath. Assoc.* 103 219–224. 12776762

[B26] Kodituwakku P. W. (2009). Neurocognitive profile in children with fetal alcohol spectrum disorders. *Dev. Disabil. Res. Rev.* 15 218–224. 10.1002/ddrr.73 19731385 PMC3104937

[B27] Korr I. M. (1991). Osteopathic research: the needed paradigm shift. *J. Am. Osteopath. Assoc.* 91:156. 10.1515/jom-1991-900210 2013534

[B28] Lanaro D. Ruffini N. Manzotti A. Lista G. (2017). Osteopathic manipulative treatment showed reduction of length of stay and costs in preterm infants: a systematic review and meta-analysis. *Medicine* 96:e6408. 10.1097/MD.0000000000006408 28328840 PMC5371477

[B29] Lange S. Rehm J. Anagnostou E. Popova S. (2018). Prevalence of externalizing disorders and autism spectrum disorders among children with fetal alcohol spectrum disorder: systematic review and meta-analysis. *Biochem. Cell Biol.* 96 241–251. 10.1139/bcb-2017-0014 28521112

[B30] Lange S. Rovet J. Rehm J. Popova S. (2017). Neurodevelopmental profile of fetal alcohol spectrum disorder: a systematic review. *BMC Psychol.* 5:22. 10.1186/s40359-017-0191-2 28645298 PMC5481937

[B31] Licciardone J. C. Kearns C. M. Hodge L. M. Bergamini M. V. (2012). Associations of cytokine concentrations with key osteopathic lesions and clinical outcomes in patients with nonspecific chronic low back pain: results from the OSTEOPATHIC trial. *J. Am. Osteopath. Assoc.* 112 596–605. 10.7556/jaoa.2012.112.9.596 22984233

[B32] Mattson S. N. Bernes G. A. Doyle L. R. (2019). Fetal alcohol spectrum disorders: a review of the neurobehavioral deficits associated with prenatal alcohol exposure. *Alcohol. Clin. Exp. Res.* 43 1046–1062. 10.1111/acer.14040 30964197 PMC6551289

[B33] Maya-Enero S. Ramis-Fernández S. M. Astals-Vizcaino M. García-Algar Ó. (2021). [Neurocognitive and behavioral profile of fetal alcohol spectrum disorder]. *An. Pediatr.* 95 208.e1–208.e9. 10.1016/j.anpede.2020.12.012 34456169

[B34] McLachlan K. Flannigan K. Temple V. Unsworth K. Cook J. L. (2020). Difficulties in daily living experienced by adolescents, transition-aged youth, and adults with fetal alcohol spectrum disorder. *Alcohol. Clin. Exp. Res.* 44 1609–1624. 10.1111/acer.14385 32472600

[B35] Ordenewitz L. K. Weinmann T. Schlüter J. A. Moder J. E. Jung J. Kerber K. (2021). Evidence-based interventions for children and adolescents with fetal alcohol spectrum disorders - a systematic review. *Eur. J. Paediatr. Neurol.* 33 50–60. 10.1016/j.ejpn.2021.02.001 34058625

[B36] Palacios J. Adroher S. Brodzinsky D. M. Grotevant H. D. Johnson D. E. Juffer F. (2019). Adoption in the service of child protection: an international interdisciplinary perspective. *Psychol. Public Policy Law* M. E. Lamb 25 57–72. 10.1186/s12913-016-1423-5 27409075 PMC4943498

[B37] Parnell Prevost C. Gleberzon B. Carleo B. Anderson K. Cark M. Pohlman K. A. (2019). Manual therapy for the pediatric population: a systematic review. *BMC Complement. Altern. Med.* 19:60. 10.1186/s12906-019-2447-2 30866915 PMC6417069

[B38] Petrenko C. L. (2015). Positive behavioral interventions and family support for fetal alcohol spectrum disorders. *Curr. Dev. Disord. Rep.* 2 199–209. 10.1007/s40474-015-0052-8 26380802 PMC4569135

[B39] Petrenko C. L. Alto M. E. (2017). Interventions in fetal alcohol spectrum disorders: an international perspective. *Eur. J. Med. Genet.* 60 79–91. 10.1016/j.ejmg.2016.10.005 27742482 PMC5205562

[B40] Ponzo V. Cinnera A. M. Mommo F. Caltagirone C. Koch G. Tramontano M. (2018). osteopathic manipulative therapy potentiates motor cortical plasticity. *J. Am. Osteopath. Assoc.* 118 396–402. 10.7556/jaoa.2018.084 29809257

[B41] Portellano Pérez J. Mateos Mateos R. Martínez Arias R. Tapia Pavón A. Granados García Tenorio M. (2009). *Cuestionario De Madurez Neuropsicológica Infantil.* (Madrid: TEA Ediciones).

[B42] Portellano-Pérez J. A. Mateos R. Martínez Arias R. Sánchez-Sánchez F. (2021). *CUMANIN-2, Cuestionario De Madurez Neuropsicológica Infantil-2.* (Madrid: TEA Ediciones).

[B43] Pruner M. Jirikowic T. Yorkston K. M. Olson H. C. (2020). The best possible start: a qualitative study on the experiences of parents of young children with or at risk for fetal alcohol spectrum disorders. *Res. Dev. Disabil.* 97:103558. 10.1016/j.ridd.2019.103558 31884315

[B44] Reid N. Dawe S. Shelton D. Harnett P. Warner J. Armstrong E. (2015). Systematic review of fetal alcohol spectrum disorder interventions across the life span. *Alcohol. Clin. Exp. Res.* 39 2283–2295. 10.1111/acer.12903 26578111

[B45] Roura S. Álvarez G. Solà I. Cerritelli F. (2021). Do manual therapies have a specific autonomic effect? An overview of systematic reviews. *PLoS One* 16:e0260642. 10.1371/journal.pone.0260642 34855830 PMC8638932

[B46] Ruffini N. D’Alessandro G. Mariani N. Pollastrelli A. Cardinali L. Cerritelli F. (2015). Variations of high frequency parameter of heart rate variability following osteopathic manipulative treatment in healthy subjects compared to control group and sham therapy: randomized controlled trial. *Front. Neurosci.* 9:272. 10.3389/fnins.2015.00272 26300719 PMC4523739

[B47] Salvador-Cruz J. Tovar-Vital D. S. Segura-Villa A. Ledesma-Amaya L. García-Anacleto A. Aguillón-Solis C. (2019). Neurological soft signs and cognitive processes in Mexican schoolchildren aged 6 to 11 years. *Act. Colom. Psicol.* 22 28–52.

[B48] Schwerla F. Daake B. Moeckel E. Resch K. L. (2021). Osteopathic treatment of infants in their first year of life: a prospective multicenter observational study (OSTINF Study). *Complement. Med. Res.* 28 395–406. 10.1159/000514413 33601373

[B49] Sjöwall D. Bohlin G. Rydell A. M. Thorell L. B. (2017). Neuropsychological deficits in preschool as predictors of ADHD symptoms and academic achievement in late adolescence. *Child Neuropsychol.* 23 111–128. 10.1080/09297049.2015.1063595 26212755 PMC5214099

[B50] Sleszynski S. L. Glonek T. (2005). Outpatient osteopathic SOAP note form: preliminary results in osteopathic outcomes-based research. *J. Am. Osteopath. Assoc.* 105 181–205. 15928337

[B51] Sleszynski S. L. Glonek T. Kuchera W. A. (1999). Standardized medical record: a new outpatient osteopathic SOAP note form: validation of a standardized office form against physician’s progress notes. *J. Am. Osteopath. Assoc.* 99 516–529. 10.7556/jaoa.1999.99.10.516 10578559

[B52] Snider K. T. Johnson J. C. Degenhardt B. F. Snider E. J. (2011). Low back pain, somatic dysfunction, and segmental bone mineral density T-score variation in the lumbar spine. *J. Am. Osteopath. Assoc.* 111 89–96. 21357494

[B53] Stade B. C. Stevens B. Ungar W. J. Beyene J. Koren G. (2006). Health-related quality of life of Canadian children and youth prenatally exposed to alcohol. *Health Qual. Life Outcomes* 4:81. 10.1186/1477-7525-4-81 17040571 PMC1617087

[B54] Standley P. R. Meltzer K. (2008). *In vitro* modeling of repetitive motion strain and manual medicine treatments: potential roles for pro- and anti-inflammatory cytokines. *J. Bodyw. Mov. Ther.* 12 201–203. 10.1016/j.jbmt.2008.05.006 19083676 PMC2622428

[B55] Tozzi P. (2015). A unifying neuro-fasciagenic model of somatic dysfunction - underlying mechanisms and treatment - Part II. *J. Bodyw. Mov. Ther.* 19 526–543. 10.1016/j.jbmt.2015.03.002 26118526

[B56] Tramontano M. Cerritelli F. Piras F. Spanò B. Tamburella F. Caltagirone C. (2020). Brain connectivity changes after osteopathic manipulative treatment: a randomized manual placebo-controlled trial. *Brain Sci.* 10:969. 10.3390/brainsci10120969 33322255 PMC7764238

[B57] Urzúa A. Ramos M. Alday C. Alquinta A. (2010). Madurez neuropsicológica en preescolares: propiedades psicométricas del test CUMANIN. *Ter. Psicol.* 28 13–25.

[B58] Van Buskirk R. L. (1990). Nociceptive reflexes and the somatic dysfunction: a model. *J. Am. Osteopath. Assoc.* 90 792–794. 10.1515/jom-1990-900916 2211195

[B59] Weyrauch D. Schwartz M. Hart B. Klug M. G. Burd L. (2017). Comorbid mental disorders in fetal alcohol spectrum disorders: a systematic review. *J. Dev. Behav. Pediatr.* 38 283–291. 10.1097/DBP.0000000000000440 28460370

[B60] Wiesman A. I. Wilson T. W. (2020). Attention modulates the gating of primary somatosensory oscillations. *Neuroimage* 211:116610. 10.1016/j.neuroimage.2020.116610 32044438 PMC7111587

[B61] World Health Organization (2012). *Benchmarks for Training in Osteopathy.* Geneva: WHO.

